# Long-Term Results in Obstructive Sleep Apnea Syndrome (OSAS) after Laser-Assisted Uvulopalatoplasty (LAUP)

**DOI:** 10.1371/journal.pone.0100211

**Published:** 2014-06-30

**Authors:** Önder Göktas, Mustafa Solmaz, Gökhan Göktas, Heidi Olze

**Affiliations:** Charité University of Berlin, CCM, Department of Otorhinolaryngology, Berlin, Germany; University of Michigan, United States of America

## Abstract

Obstructive sleep apnea syndrome (OSAS) is a serious disease. The etiology of and optimum therapy options for this disorder have been much discussed and have been the subject of many publications. One much discussed therapy option is laser-assisted uvulopalatoplasty (LAUP). Despite conflicting opinions and guidelines which recommend that it should not be used, it remains in use. Patients who had previously undergone this procedure were invited for follow-up appointments, at which they were asked to complete a questionnaire, underwent an ENT examination and underwent sleep laboratory analysis using a portable sleep lab device. The average time since LAUP treatment was 11 years. The cohort comprised 25 patients. The average preoperative apnea-hypopnea-index (AHI) score was 25.25/h; the average postoperative AHI score 23.62/h. Closer examination of our data enabled us to identify 10 responders (40%) and 15 non-responders (60%). 12% (3/25) of non-responders experienced either no reduction in their AHI score or an increase compared to their preoperative AHI score of less than 5/h. In the remaining 48% (12/25), AHI increased by more than 5/h compared to the preoperative figure. Our questionnaire showed that 40% (10/25) of patients suffered from dry mouth and 20% (5/25) from foreign body sensation. The data led us to conclude that laser-assisted uvulopalatoplasty can indeed result in a reduction in AHI score comparable to other mucosal resection methods. Also in common with these methods, the efficacy of the therapy reduces with time and the procedure carries a high risk of bringing about an increase in the patient's AHI score.

## Introduction

Obstructive sleep apnea syndrome (OSAS) is the most common sleep-related breathing disorder. The prevalence of OSAS in the male population is believed to be 4% and in the female population 2%. Its incidence is estimated to be 15% for men and 8.2% for women [1].

**Figure 1:**
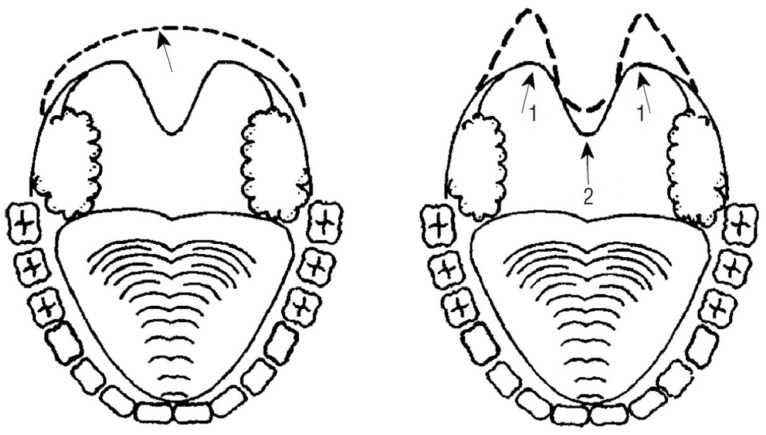
Schematic representation of laser-assisted uvulopalatoplasty (LAUP). The left-hand diagram shows complete resection of the uvula, the right-hand diagram shows preservation of the uvula by means of para-uvular incisions [6].

Laser-assisted uvulopalatoplasty (LAUP) is a much discussed option for surgical treatment of OSAS [2]–[4]. LAUP was first described in 1990 and is a mucosal resection technique [5]. In contrast to uvulopalatopharyngoplasty (UPPP), LAUP does not require the insertion of stitches and can be performed as an outpatient procedure. Like UPPP, LAUP has gone through a number of iterations over time and has now been in use for the treatment of snoring and OSAS for several years. The goal of laser-assisted uvulopalatoplasty is enlargement of the airspace in the oropharyngeal region by means of resection and tightening using a C0_2_ (carbon dioxide) or Nd:YAG (neodymium-doped yttrium aluminum garnet) laser. Scar formation plays a key role in determining the success of the operation. The procedure involves making bilateral vertical incisions in the para-uvular region of the soft palate. The incisions run 1–2 cm (depending on patient anatomy) laterally toward (but do not attain) the palatal vault, and cranially to the edge of the mucosa and muscle. The uvula is then resected. In an alternative method, after making the incisions described above, only a portion of the uvula is resected in the para-uvular region, such that the base of the uvula is retained (Figure 1) [6]. Neither technique involves tonsillectomy or the use of stitches [3], [7]–[9].

The aim of this study is to evaluate long-term treatment outcomes in patients with OSAS and to evaluate LAUP as a surgical treatment. The study presents results from long-term evaluation of patients treated with LAUP. The average time elapsed since treatment was more than 11 years.

We also used a questionnaire to look for the occurrence of known postoperative surgical complications of and risks associated with laser-assisted uvulopalatoplasty identified in previous studies.

## Methods

### Patient selection

All patients were treated between 1997 and 2002 in the department for otorhinolaryngology, head and neck surgery at the University Clinic Berlin Campus Charité Mitte. The patients had previously undergone polysomnography testing in a sleep laboratory for suspected sleep-related breathing disorders (SRBD). All patients were found to have some degree of OSAS or snoring. After considering a full range of treatment options and obtaining the patient's consent, LAUP was selected as the most appropriate treatment option.

Patients treated using other surgical techniques, such as uvulopalatopharyngoplasty (UPPP), were not included in the study. A total of 25 patients were included in the study.

Patients underwent a clinical examination, their current and preoperative body mass indices (BMI) were determined and the researcher went through and filled out a questionnaire concerned snoring behavior with the patient. Patients were asked to subjectively classify their preoperative, immediate postoperative and current snoring activity on a scale of 0 to 10 (0 =  no snoring, 10 =  continuous snoring throughout the night).

Patients were also asked about side effects, such as a foreign body sensation, dry mouth, nasal voice, general change in voice, daytime fatigue, concentration difficulties or a tendency to fall asleep.

After completing the questionnaire, patients were asked to assess their satisfaction with LAUP as a therapy method for their disorder and the postoperative outcome on a scale of 0–10 (0 =  completely dissatisfied, 10 =  completely satisfied).

For the sleep laboratory study, the patients were provided with a portable device, which is mounted on the chest using a belt. The device records throughout the night or period of sleep. Polygraphic data from the device was extracted and evaluated by a specialist. The device used for outpatient sleep apnea screening was the Sleep Doc Porti 5 manufactured by Boppel.

All patient data was de-identified and analyzed in anonymised form. Examinations were not carried out and data was not collected specifically for this study. All patients have given written consent for their data to be published.

### Statistical analysis

Statistical calculations and analysis were performed using SPSS version 20 from IBM. Tables, charts, and graphics were created using SPSS version 20 and Excel 2010 from Microsoft.

Statistical evaluation was carried out using the Wilcoxon test for non-parametric paired samples. Preoperative apnea hypopnea index (AHI) scores were compared with long-term postoperative AHI scores and the mean and standard deviation determined and tested for statistical significance.

## Results

The study cohort comprised 22 men and 3 women. Their average age was 63.32 years. Their mean BMI at the time of surgery was 30. Follow-up examinations were carried out on average 134 months (≥11 years) after laser-assisted uvulopalatoplasty treatment. The mean preoperative AHI value was 25.42/h, the mean AHI value after ≥11 years 23.62/h.

Therapy outcomes were evaluated based on success metrics proposed by Sher *et al*. (Table 1) [10]. Treatment of patients with obstructive sleep apnea syndrome is considered successful if the postoperative AHI score is at least 50% lower than the preoperative score and less than 20/h.

**Table 1:** Overview of classification criteria, showing designations, abbreviations and definitions.

Designation	Abbreviation	Definition
Cure	C	AHI <5/h.
Responder	R	Reduction in AHI of at least 50% and an AHI ≤20/h.
Non-responder	NR	Does not meet the criteria given by Sher *et al*. and AHI has increased by <5/h.
Non-responder with deterioration	NR with deterioration	Does not meet the criteria given by Sher *et al*. and AHI has increased by ≥5/h

To improve the way the results represent the degree of severity, in assessing the questionnaire on snoring behavior, subjective snoring behavior scores were subdivided into the categories ‘rarely’ (0–2), ‘occasionally’ (3–5), ‘frequently’ (6–8), and ‘always’ (9–10). Snoring behavior was only considered to have improved or worsened if the severity category had changed. Where the snoring score had remained the same or had changed but remained within the same severity category, the patient was evaluated as have experienced no change in snoring behavior.

Similarly, to improve representation and evaluation, patient satisfaction scores (0–10) were divided into ‘completely dissatisfied’ (0–2), ‘somewhat dissatisfied’ (3–5), ‘fairly satisfied’ (6–8), and ‘completely satisfied’ (9–10). The results, including BMI, AHI, and patients’ subjective reporting of their snoring behavior prior to LAUP, immediately after LAUP, and at the time of the survey are shown in Table 2.

**Table 2:** Polysomnograpy results showing preoperative and postoperative severity category, difference, means, age, preoperative BMI, time in months since LAUP and pre and postoperative AHI.

Patient ID	Months since LAUP	BMI (KG/[height in m]^2^)		OSA severity: AHI/h*		Subjective snoring score**		
		Before surgery	After surgery	Before surgery	After surgery	Before surgery	After surgery	Current
1	128	38.6	34.5	11.4	36.7	10	9	9
2	122	33.9	31.6	57.3	1.7	10	1	1
3	116	25.8	27.4	27.9	9	10	7	5
4	71	31.9	31.5	13.6	4.8	10	0	3
5	127	36.4	38.3	2.7	3	9	9	9
6	127	23.2	23.2	10.1	39	10	3	3
7	114	30.1	31.7	36	75	7	6	5
8	178	37	40.1	8.1	41	10	5	5
9	164	27.7	24.2	38.6	51	10	3	3
10	134	29.3	29.3	9.7	23	10	10	10
11	125	26.1	29.4	55	13	10	7	10
12	207	32.1	32.1	1.4	20	10	3	10
13	126	27	30.4	30.6	0.8	10	10	10
14	125	30.9	34	20.4	39.8	10	7	10
15	122	23.5	24.9	43.7	13	8	1	5
16	156	24.6	27.4	31.4	4.7	10	8	8
17	152	30.1	26.1	12.5	1.7	8	5	5
18	112	31	32.5	30.6	26.8	10	7	8
19	119	27.7	32.5	62	48	10	10	10
20	74	33.2	33.2	40.7	1.7	10	1	1
21	123	32.4	29.3	10.3	4.2	8	2	5
22	169	36.8	36.8	46.9	36.9	10	5	5
23	169	24.8	27.3	21.6	33.7	8	0	10
24	170	27.5	29	11.7	23	10	4	5
25	126	30.7	29.2	14.6	39	10	9	9
**Mean Values**	**134.24 months**	**BMI before surgery 30.092**	**BMI after surgery 30.636**	**AHI before surgery 25.425/h**	**AHI after surgery 23.62/h**	**Before surgery: always (9.52)**	**After surgery: occasional (5.28)**	**Current: occasional (6.56)**

* OSA severity: AHI <5/h  =  normal; 5–20/h  =  mild; 21–40/h  =  moderate, >40/h  =  severe.

** Subjective snoring score: 0–2 =  rare; 3–5 =  occasional; 6–8 =  often; 9–10 =  always.

Before LAUP, 10 of the 25 patients (40%) were diagnosed with mild obstructive sleep apnea syndrome (AHI  = 5–20/h), 7 (28%) with moderate obstructive sleep apnea syndrome (AHI  = 21–40/h), 6 (24%) with severe obstructive sleep apnea syndrome (AHI ≥40/h) and 2 (8%) with no obstructive sleep apnea syndrome (AHI ≤5/h).

Analysis of the polysomnography results showed that, more than 11 years postoperatively, 4 of the 25 patients (16%) were suffering from mild OSAS (AHI 5–20/h), 9 (36%) from moderate OSAS (AHI 21–40/h), 4 (16%) from severe OSAS (AHI ≥40/h) and 8 (32%) were not suffering from OSAS (AHI ≤5/h).

Analysis of individual cases reveals that, in the more than 11 years since the operation, 3 patients (12% non-responders) have not experienced a significant deterioration in either their OSAS severity category or AHI and 12 patients (48% non-responders with deterioration) have experienced such a deterioration. An improvement was detected in 3 patients (12% responders) and 7 patients (28% cure) were cured of OSAS.

Prior to LAUP, 20% (5) of the patients were within the normal weight range, 40% (10) overweight and 40% (10) obese. The current BMIs, more than 11 years postoperatively, reveal that 12% (3) of the patients are within the normal weight range, 40% (10) remain overweight and 48% (11) are now obese.

It is notable that all of the patients with clinical symptoms of OSAS preoperatively snored, with 20 of them stating that they always snored and 5 stating that they often snored.

Immediately after laser-assisted uvulopalatoplasty, 6 patients reported they always snored and another 6 reported snoring frequently. Of the remaining 13 patients, 7 reported snoring only occasionally and 6 snoring rarely. At the time of the survey, 10 patients reported that they always snore, 2 that they snore often, 11 patients reported snoring only occasionally during a night's sleep, and 2 patients reported that they now snore only rarely.

Table 3 shows patient responses to questions on postoperative complications and side effects.

**Table 3:** Summary of patient responses in postoperative complications/side effects survey.

Patient ID	Foreign body sensation	Dry mouth	Nasal and/or change in voice	Daytime tiredness	Difficulty concentrating	Daytime sleepiness	Satisfaction with LAUP (0–10)*
1	no	no	no	no	no	no	5
2	no	no	no	yes	no	no	10
3	no	no	no	no	no	no	6
4	no	no	yes	no	no	no	7
5	no	no	no	no	no	no	0
6	yes	no	no	no	yes	no	10
7	no	yes	no	no	no	no	5
8	no	yes	no	no	no	no	10
9	no	no	no	no	no	no	8
10	no	no	yes	yes	no	no	0
11	no	yes	no	no	no	no	2
12	no	yes	no	yes	no	no	2
13	no	yes	no	yes	no	no	2
14	no	yes	no	no	no	no	1
15	yes	no	no	no	no	no	7
16	no	no	no	no	no	no	7
17	no	no	no	no	no	no	7
18	no	yes	no	yes	yes	yes	7
19	yes	yes	no	yes	no	no	0
20	no	no	no	no	no	no	7
21	yes	no	no	no	no	no	6
22	yes	no	no	no	no	no	6
23	no	yes	no	no	yes	no	2
24	no	no	no	yes	no	no	8
25	no	yes	no	no	no	nein	3
**Total of 25**	**Yes 20%; No 80%**	**Yes 40%; No 60%**	**Yes 8%; No 92%**	**Yes 28%; No 72%**	**Yes 12%; No 88%**	**Yes 4%; No 96%**	**Mean value: 5.12**

*0–2 =  completely dissatisfied; 3–5 =  somewhat dissatisfied; 6–8 =  fairly satisfied; 9–10 =  completely satisfied.

In their responses to our questionnaire, 32% (8) of patients reported being completely dissatisfied, 12% (3) were somewhat dissatisfied, 44% (11) were fairly satisfied and 12% (3) were completely satisfied with the treatment outcome.

## Discussion

The goal of treatment for obstructive sleep apnea syndrome is to normalize breathing and sleeping during the night or sleep.

Polysomnography of our 25 patients an average of more than 11 years after LAUP found that 28% (7) of the patients had been cured of their OSAS and that 12% (3 responders) had experienced a reduction in their apnea hypopnea index (AHI) score of more than 50% and had an AHI score ≤20/h. No change was observed in 12% (3 non-responders) of the patients and deterioration was observed in 48% (12 non-responders with deterioration) of the patients. Comparing the mean preoperative (25.95/h) and postoperative AHI scores (23.62/h) an average of more than 11 years postoperatively suggests that laser-assisted uvulopalatoplasty does not result in a statistically significant successful long-term therapeutic outcome.

In 1994, Kamami published results from 46 patients with obstructive sleep apnea syndrome treated between 1988 and 1993 using his laser-assisted uvulopalatoplasty technique [5], [11] and recommended its use with patients suffering from snoring or OSAS [11].

In 2002 Finkelstein *et al*. published polysomnography results from 26 patients obtained from sleep laboratory studies carried out on average 12 months after LAUP. Their mean respiratory disturbance index (RDI) score was 29.6/h preoperatively, falling to 25/h postoperatively. 8 (31%) of these patients were found to have been cured after surgery, 7 (26%) of these patients experienced an improvement of less than 50% compared to the baseline value, no significant change was observed in 3 (12%) of the patients and 8 (31%) of the patients experienced a deterioration following surgery [12]. In 2003, Berger *et al*. from the same research group published results from 25 patients. 60% of these patients experienced deterioration. The mean RDI was 25.3/h preoperatively, increasing to 33.1/h postoperatively [13].

In 2003, Ferguson *et al*. published results from the first controlled randomized trial. The trial encompassed 45 patients, of whom 21 were treated with laser-assisted uvulopalatoplasty. Postoperative polysomnography an average of 15 months after surgery showed a favorable treatment outcome in 5 (24%) patients, with treatment classified as a failure in the remaining 16 (76%) patients [14].

Average preoperative and postoperative AHI scores from these individual studies clearly do not provide a basis on which to recommend the use of LAUP for treating patients with OSAS.

In our patients we found no significant change in BMI between surgery and the follow-up examination. It is important to note that the aging process and slackening of tissues associated with it can cause further obstruction and is likely to be an important factor. The question this study aims to address is not whether patients with obstructive sleep apnea syndrome should be treated surgically, but whether laser-assisted uvulopalatoplasty offers short and long-term benefits. Whether therapy should be sought for OSAS is not in question, since its consequences can be fatal. Due consideration also needs to be given to alternative soft palate procedures such as uvulopalatopharyngoplasty (UPPP), which are more efficient than LAUP and are recommended in both German and American guidelines for treatment of obstructive sleep apnea syndrome [2], [3].

As discussed above, LAUP is considered to be contraindicated for the treatment of obstructive sleep apnea syndrome [3], [4]. The average success rate for LAUP is about 50%. This is no worse than UPPP, which has an average success rate of about 40–55% [6]. In our view, the key argument against the use of laser-assisted uvulopalatoplasty to treat patients with obstructive sleep apnea syndrome is not the figures for responders, but rather the frequency with which the procedure results in a postoperative increase in patient AHI scores.

In our study 48% of patients were classified as non-responders with deterioration. This figure, which is significantly higher than the mean value of 29%, may be explained by the fact that patients were followed up more than 11 years after treatment. This finding would appear to suggest that the effects of treatment diminish over time.

Comparing results from previous studies published elsewhere objectively with the results of this study, treatment of patients with obstructive sleep apnea syndrome with LAUP does appear to deliver short-term benefits, but there is also a high risk that the treatment will deliver no improvement and an unacceptably high risk that treatment may even have the opposite effect to that intended. No factors predictive for success or failure of the therapy were found. Whether the therapy is successful appears to be largely a matter of chance and appears to depend on individual postoperative wound healing/scar formation and thus on the size of the oropharyngeal and velopharyngeal spaces produced. In addition, our study shows that the therapeutic effect decreases over an extended period of time and with the aging process.

In our study of patients more than 11 years postoperatively, the treatment was found to be objectively successful in 40% of patients and unsuccessful in 60% of patients. Our questionnaire, however, found that 60% of patients remained satisfied with the treatment outcome and only 40% dissatisfied. The objective results, in contrast to the subjective results, also tally with patients' subjective responses to questions on daytime fatigue (72% said no), concentration difficulties (88% said no) and tendency to fall asleep (96% said no) – typical symptoms of obstructive sleep apnea syndrome which were not reported by most of our patients.

Publications in this area are frequently criticized for in some cases large variations between results, an observation which has been much discussed. This is the result of data being collected shortly after or less than 1 year after surgery, providing little information on whether the effects of surgery are persistent. The subjective results from our 25 patients suffering from snoring showed an improvement or cure rate of 72% in the immediate postoperative period (daytime fatigue) and 60% an average of 11 years later. Based on these results, it can be concluded that LAUP must be taken seriously as an option for treatment of patients suffering from snoring. Over time, however, the effects of treatment appear to diminish, though a persistent long-term benefit is observed in patients' subjective reporting of snoring.

As with any therapy, laser-assisted uvulopalatoplasty can also give rise to postoperative complications. The only side effect reported in the initial paper on this technique was postoperative sore throat treatable with analgesia [5]. With increased use of the technique for treating patients with snoring and obstructive sleep apnea syndrome, independent publications over a period of several years have reported an increase in the incidence of postoperative complications. In addition to postoperative pain, foreign body sensation was reported in 8–25% of patients, voice changes in 0–17.2%, and an increase in the incidence of dry mouth of between 16 and 42% [12], [15], [16]. 20% of our patients suffer from foreign body sensation in their throat and only 8% of patients suffer from persistence of nasal language or speech changes.

Looking at our data overall, we conclude that laser-assisted uvulopalatoplasty (LAUP) can indeed result in a reduction in AHI score comparable to that achieved with other mucosal resection methods, but that its effects diminish with time. There is also a high risk of achieving the opposite effect. We would not therefore recommend using this procedure to treat patients with OSAS.
